# Phosphatase and Tensin Homology Deleted on Chromosome 10 Inhibitors Promote Neural Stem Cell Proliferation and Differentiation

**DOI:** 10.3389/fphar.2022.907695

**Published:** 2022-06-14

**Authors:** Xiaojiang Liu, Yiqiu Cui, Jun Li, Cheng Guan, Shu Cai, Jinrong Ding, Jianhong Shen, Yixiang Guan

**Affiliations:** ^1^ Department of Neurosurgery, Affiliated Haian Hospital of Nantong University, Nantong, China; ^2^ Department of Neurosurgery, Affiliated Hospital of Nantong University, Nantong, China

**Keywords:** Pten, mTOR, neural stem cells, proliferation, differentiation

## Abstract

Phosphatase and tensin homology deleted on chromosome 10 (PTEN) is a tumor suppressor gene. Its encoded protein has phosphatase and lipid phosphatase activities, which regulate the growth, differentiation, migration, and apoptosis of cells. The catalytic activity of PTEN is crucial for controlling cell growth under physiological and pathological conditions. It not only affects the survival and proliferation of tumor cells, but also inhibits a variety of cell regeneration processes. The use of PTEN inhibitors is being explored as a potentially beneficial therapeutic intervention for the repair of injuries to the central nervous system. PTEN influences the proliferation and differentiation of NSCs by regulating the expression and phosphorylation of downstream molecular protein kinase B (Akt) and the mammalian target of rapamycin (mTOR). However, the role of PTEN inhibitors in the Akt/mTOR signaling pathway in NSC proliferation and differentiation is unclear. Dipotassium bisperoxo (picolinoto) oxovanadate (V) [bpv(pic)] is a biologically active vanadium compound that blocks PTEN dephosphorylation and suppresses its activity, and has been used as a PTEN lipid phosphatase inhibitor. Here, bpv(pic) intervention was found to significantly increase the number of rat NSCs, as determined by bromodeoxyuridine staining and the cell counting kit-8, and to increase the percentage of neurons undergoing differentiation, as shown by immunofluorescence staining. Bpv(pic) intervention also significantly increased PTEN and mTOR expression, as shown by real-time PCR analysis and western blotting. In conclusion, PTEN inhibitor bpv(pic) promotes the proliferation and differentiation of NSCs into neurons.

## Introduction

As an anti-oncogene with dual specific phosphatase activity, phosphatase and tensin homology deleted on chromosome 10 (PTEN) has become a research hotspot in recent years. It plays an important role in a variety of diseases, including cancer, liver disease (Ikeda et al., 2020; Chen et al., 2021), and diabetes (Lu et al., 2021), where it is involved in cell migration, proliferation, differentiation, apoptosis, and metabolism (Yamada and Araki, 2001; Hamada et al., 2005; Salmena et al., 2008; Chow and Salmena, 2020).

PTEN mainly catalyzes the conversion of phosphatidylinositol trisphosphate (PIP3) to phosphatidylinositol biphosphate (PIP2) by inhibiting the classical phosphatidylinositol 3 kinase (PI3K)-serine/threonine kinase (Akt) signaling pathway (Song et al., 2005). When PI3K receives signals from tyrosine kinase and G protein-coupled receptors, activated PI3K converts PIP2 to PIP3, and reduces PIP3 to PIP2. PIP3 then binds to the N-PI3KPH domain of downstream Akt, which is transferred from the cytoplasm to the cell membrane (Park et al., 2010).

With the assistance of 3-phosphoinositol-dependent protein kinase 1, PIP3 activates Akt by phosphorylating its threonine phosphorylation site (Thr308) or serine phosphorylation site (Ser473). Activated Akt then activates mammalian target of rapamycin (mTOR). The PI3K/Akt/mTOR signaling pathway activates and regulates cell proliferation, differentiation, and migration (Jung et al., 2021). The pathway is also involved in the repair and regeneration of central nerve injuries, as shown by PTEN gene knockout using a PTEN inhibitor or small interfering RNA which accelerated the growth of axons at the injured site (Lu et al., 2020)^.^ Although PTEN is not required to determine cell fate in the central nervous system (CNS), it was shown to function in NSC differentiation, where its expression changes dynamically. PTEN expression begins in the late stages of mouse CNS development and peaks in adulthood. It is widely expressed in the brain of adult mice, especially in neurons (Li et al., 2020; Yu et al., 2020).

mTOR is an important signaling molecule in the PTEN signaling pathway, which regulates pentameric neuronal ASH2-like, histone lysine methyltransferase complex subunit at the transcriptional level (Nguyen and Anderson, 2018). Consequently, it affects neuronal differentiation and directional axonal outgrowth (Jia et al., 2021). PI3K/AKT/mTOR signaling was shown to regulate neuronal cell maturation and differentiation, while Park (Park et al., 2008) reported regeneration of the optic nerve after PTEN knockdown following the reactivation of PI3K/Akt/mTOR signaling. PTEN also regulates neuronal apoptosis, proliferation, renewal, and differentiation, and inhibits neuronal regeneration by inhibiting transduction of the PI3K/AKT signaling pathway. Thus, inhibiting PTEN promotes the survival and differentiation of NSCs. 

Vanadium and vanadium peroxide compounds are widely used as general inhibitors of protein tyrosine phosphatase, especially bisperoxovanadium compounds which include dipotassium bisperoxo (picolinoto) oxovanadate (V) [bpV(pic)] (vanadium diperoxys 5-hydroxypyridine). Bpv(pic) is a specific inhibitor of PTEN that promotes neural stem cell (NSC) proliferation and differentiation *in vitro* and *in vivo*, with no significant effect on cell survival (Guan et al., 2021). Together, these findings suggest that PTEN plays an important role not only in peripheral nerve damage but also in the repair and regeneration of central nerve injury.

In this study, we examined the role of a PTEN inhibitor in NSC proliferation and differentiation. We found that inhibiting PTEN expression decreased neuronal proliferation and differentiation through the activation of PI3K/Akt/mTOR signaling. Our findings enhance our understanding of the mechanism of NSC differentiation during neurogenesis.

## Materials and Methods

### Cell Lines and Reagents

Sixteen-day-old pregnant SD rats (Guan et al., 2015) were provided by the Laboratory Animal Center of Nantong University. This study was conducted in accordance with the recommendations of the National Institutes of Health Laboratory Animal Care and Use Guidelines. The isolated fetal rat cerebral cortex was removed under aseptic conditions, meninges were stripped in Dulbecco’s modified Eagle medium (DMEM) containing 0.25% trypsin for 10 min, and the cell suspension was obtained in DMEM containing 5% horse serum and 10% fetal bovine serum (Gibco, Grand Island, NY, United States) at a density of 1 × 10^6^ cells/ml. Cells were then cultured at 37°C with 5% CO_2_ in DMEM supplemented with neurobasal neuron-specific medium (Gibco) containing 1% B-27 supplement and 0.25% l-Glutamine.

### Proliferation of NSCs After bpv(pic) Intervention

After harvesting, the second generation of NSCs was seeded into 24-well plates at a density of 5 × 10^4^ cells/mL. Bpv(pic) (ATCC, Manassas, VA, United States) was added to the intervention group at a final concentration of 200 nmol/L (Thellung et al., 2019). NSCs were cultured for 5–7 days at 37°C with 5% CO_2_, then the number of cells was determined using the cell counting kit-8 (CCK-8; Abcam) and compared between the two groups. Briefly, cell proliferation was measured by adding 100 µL DMEM/F-12 and 10 µL CCK-8 reagent to each plate, and incubating for 8 h at 37°C with 5% CO_2_. The absorbance at 425 nm was then measured using the Multiskan MK33 microplate reader (Thermo Electron Corporation, Shanghai, China). Bromodeoxyuridine (BrdU) solution was also added to the intervention group at a final concentration of 5 μmol/L to stain proliferating neonatal neurons which were observed using an Olympus IX71 microscope.

### NSC Differentiation

NSCs were inoculated at a density of 5 × 10^4^ cells/ml into 24-well plates with polylysine-coated glass slides in differentiation solution (DMEM/F-12 supplemented with 1% fetal bovine serum) which was changed after 2 h (Guan et al., 2015). Bpv(pic) was added to the intervention group at a final concentration of 200 nmol/L, and all cells were cultured for a further 7 days. Cells were then incubated with the following primary antibodies at 4°C for 16 h: rabbit anti-rat βIII tubulin antibody (diluted 1:1000; Abcam), mouse anti-rat glial fibrillary acidic protein (GFAP) antibody (diluted 1:1000; Abcam), and rabbit anti-rat receptor interacting protein (RIP) antibody (diluted 1:1000; Abcam). They were then incubated with goat anti-rabbit IgG H&L (Alexa Fluor^®^ 594) (diluted 1:1000; Abcam) secondary antibodies at 20°C for 2 h. DNA was stained by immediately incubating the slides in 4′,6-diamidino-2-phenylindole (0.2 mg/ml) for 2 min. Slides were stored in the dark at 4°C, then six fields of view per slide were randomly selected. The percentage of positively staining cells in each field was calculated under an Olympus IX71 microscope, and the average value was compared between control and intervention groups.

### Real-Time PCR Analysis

Total RNA was extracted using TRIzol reagent (Invitrogen, Carlsbad, CA, United States), then reverse-transcribed into cDNA using the Omniscript RT Kit (Qiagen) according to the manufacturer’s instructions. PCR was carried out using the following conditions: 95°C for 2 min, then 30 cycles of 95°C for 15 s, 54°C for 30 s, and 72°C for 1 min (Guan et al., 2015). Primer sequences were: mTOR-F: 5′-AGG​AGG​GAC​GTT​TGC​TCA​GA-3′ and mTOR-R: 5′-TCC​CTC​ACT​GAA​CAC​AGC​AG-3′; PTEN-F: 5′-ACC​AGG​ACC​AGA​GGA​AAC​CT-3′ and PTEN-R: 5′-TTT​GTC​AGG​GTG​AGC​ACA​AG-3′; and β-actin-F: 5′-AGG​CAT​CCT​GAC​CCT​GAA​GTA​C-3′ and β-actin-R: 5′-TCT​TCA​TGA​GGT​AGT​CTG​TCA​G-3′.

### Western Blotting

Membranes were incubated with primary antibodies against β-actin (diluted 1:3000; Abcam), PTEN (diluted 1:1000; Abcam), and mTOR (diluted 1:1000; Abcam).

### Statistical Analysis

All assays were performed in duplicate a total of three times. Data are expressed as the mean ± SEM, and were analyzed by the Student’s t-test and one-way analysis of variance. SPSS v. 17.0 statistical software was used for analysis, and *p* values ≤ 0.05 were considered statistically significant.

## Results

### NSCs Self-Renewed and Proliferated

**FIGURE 1:**
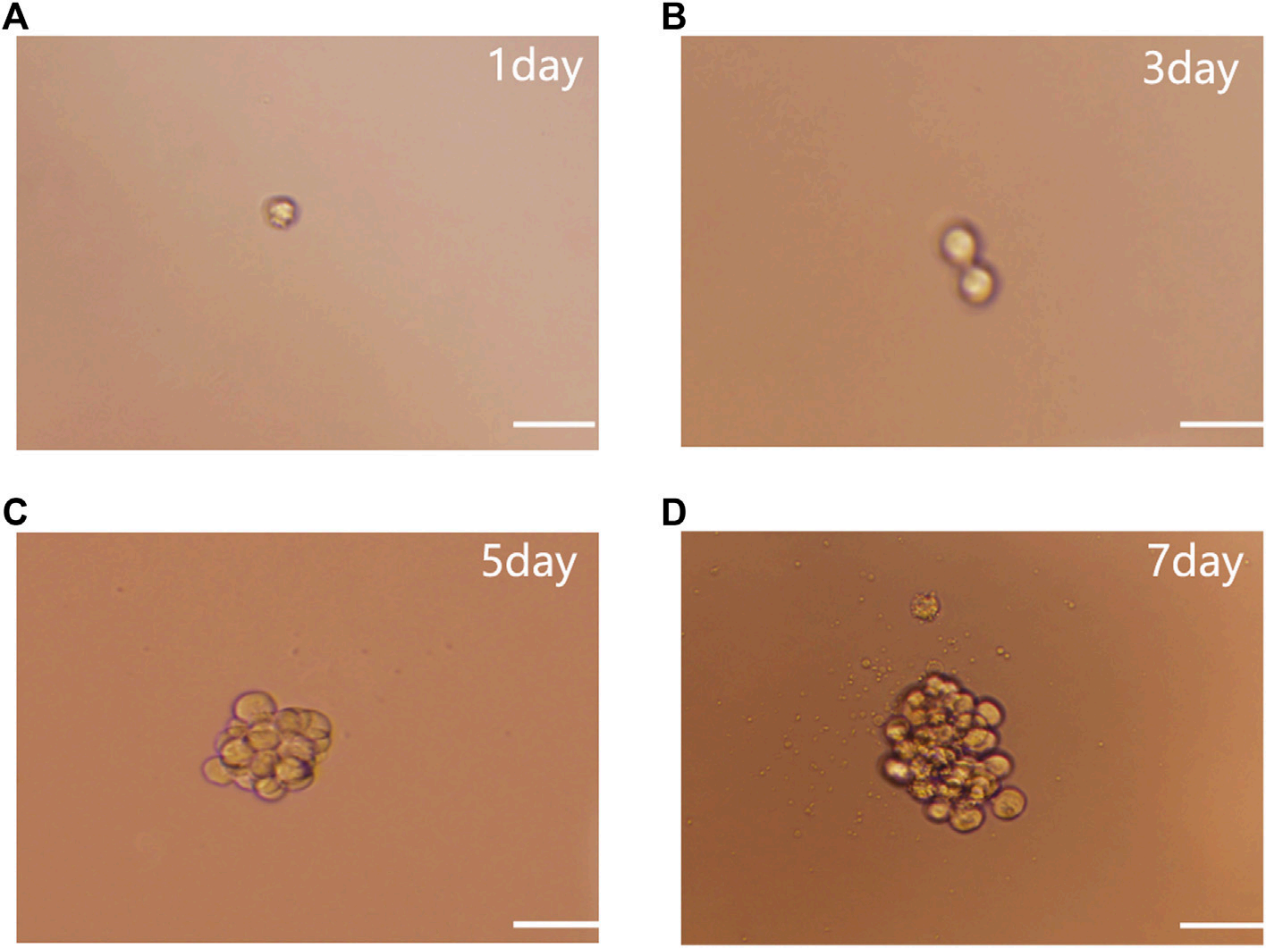
NSC single cell cloning. **(A)** Single cell culture. **(B)** After 3 days culture. **(C)** After 5 days culture. **(D)** Proliferation to form NSCs after 7 days subculture. Scale bar: 100 µm.

Single-cell cloning experiments showed that individual NSCs (Figure 1A) divided after 3 days (d) of culture (Figure 1B), exhibited colonies of 15–28 cells after 5 days (Figure 1C), and proliferated to form a colony of around 50 cells after 7 days (Figure 1D). This suggests that colony formation occurred through the self-renewal and proliferation of NSCs rather than the aggregation of individual NSCs.

### Bpv(pic) Promoted NSC Proliferation

**FIGURE 2:**
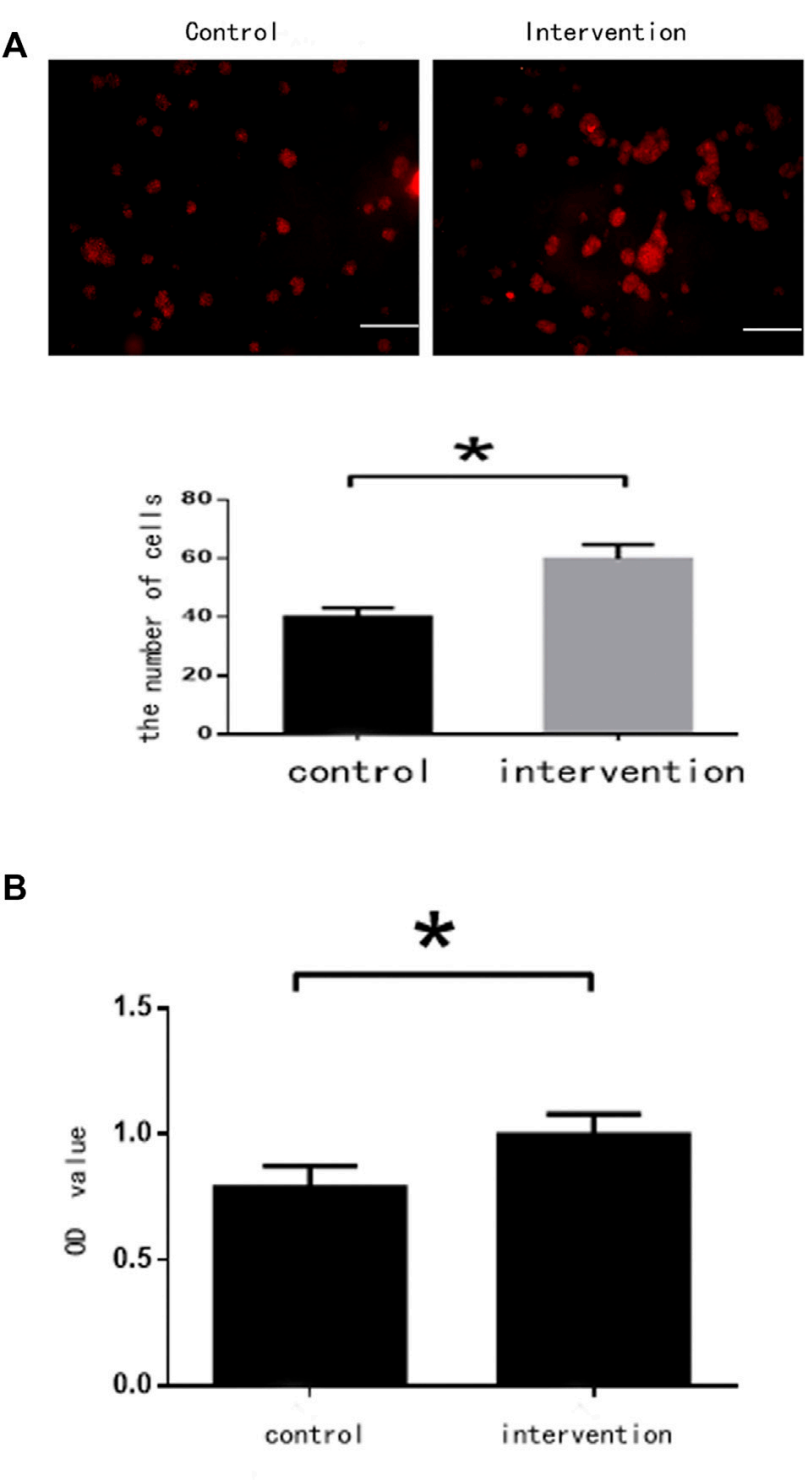
Cell assessments 5 days after bpv(pic) intervention. **(A)** Cells after staining with BrdU. Scale bar: 100 µm. **(B)** Cell proliferation as detected by the CCK-8 assay.*p < 0.05.

BrdU staining showed that the number of NSCs in the intervention group (65 ± 6 cells) was significantly higher than in the control group (42 ± 5 cells) (*p* < 0.05) (Figure 2A). Absorbance values were 0.997 ± 0.085 and 0.788 ± 0.083 for the intervention and control groups, respectively. The CCK-8 assay found that bpv(pic) significantly inhibited the proliferation of the intervention group compared with the control (*p* < 0.05). These data together suggest that bpv(pic) promoted the proliferation of NSCs (Figure 2B).

### Bpv(pic) Promoted the Differentiation of NSCs Into Neurons and Inhibited Their Differentiation Into Glial Cells

**FIGURE 3:**
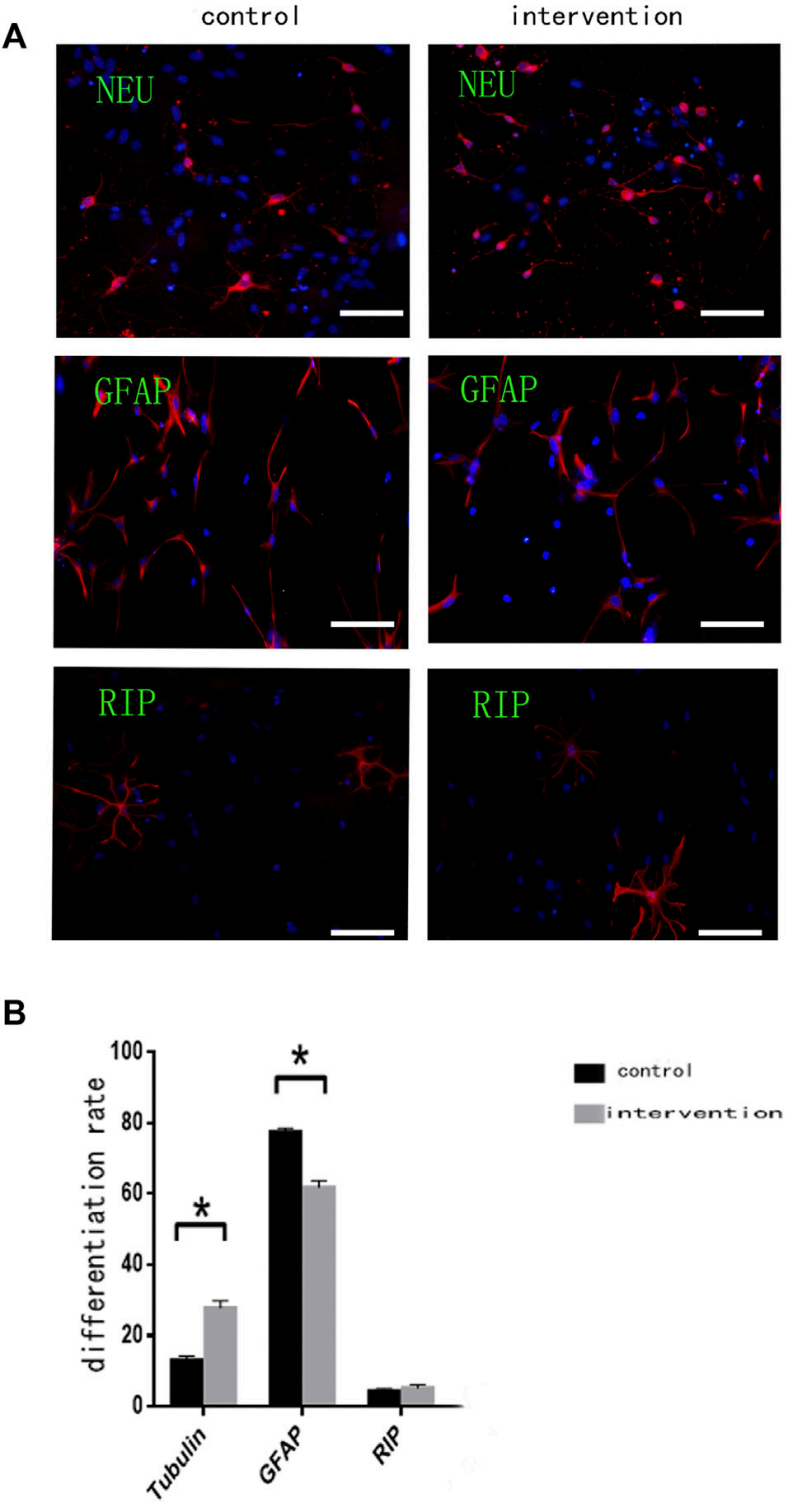
Bpv(pic) modulates the differentiation of NSCs. **(A)** Immunofluorescence using anti-β-Tubulin III, anti-GFAP, and anti-RIP antibodies after bpv(pic) intervention. Scale bar: 100 µm. **(B)** Statistical analysis of immunofluorescence. *p < 0.05.

Immunofluorescence staining (Figure 3A) with anti-βIII tubulin, anti-GFAP, and anti-RIP antibodies showed that the percentage of NSCs differentiating (Figure 3B) into neurons was significantly higher in the intervention group (27.860 ± 1.927%) than in the control group (13.120 ± 1.130%) (*p* < 0.05). Moreover, the percentage of differentiated glial cells was significantly lower in the intervention group (61.900 ± 1.840%) than in the control group (77.520 ± 1.035%) (*p* < 0.05). Some NSCs differentiated into oligodendrocytes, but there was no significant difference in the percentage of these between the two groups (*p* > 0.05).

### Bpv(pic) Enhanced the Expression of mTOR and PTEN in NSCs

**FIGURE 4:**
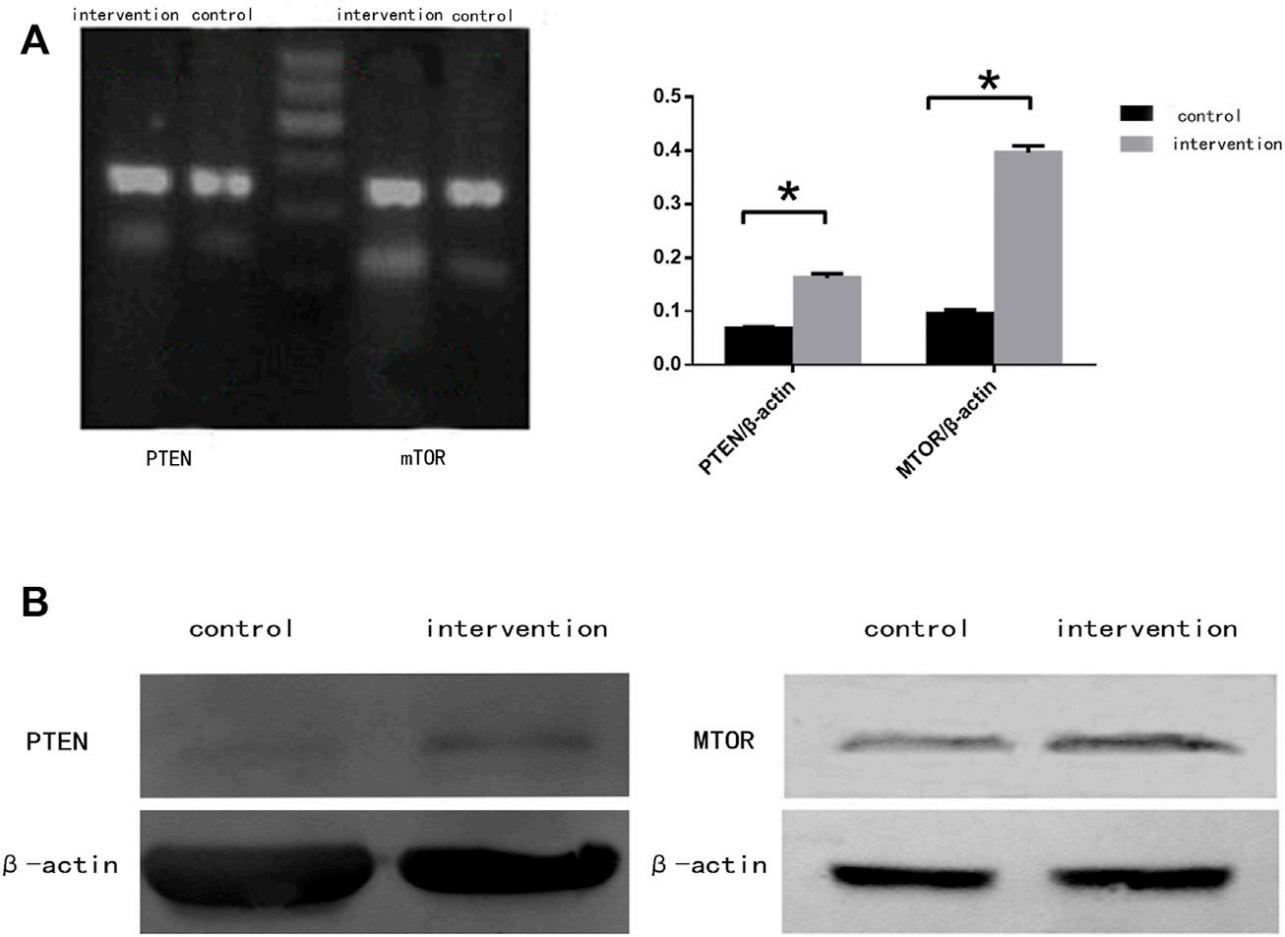
mTOR and PTEN expression in NSCs 5 days after bpv (pic) intervention. **(A)** mRNA expression of mTOR and PTEN in rat NSCs. **(B)** Protein expression of mTOR and PTEN in rat NSCs. β-actin was used as a loading control. *p < 0.05.

RT-PCR (Figure 4A) and western blotting (Figure 4B) were used to detect the expression of mTOR and PTEN at mRNA and protein levels, respectively. We observed significantly increased expression of mTOR and PTEN in the intervention group compared with the control group (*p* < 0.05), with a greater increase seen in mTOR expression.

## Discussion

Nerve regeneration and repair play important roles in nerve function recovery. NSCs are key cells in these processes because of their potential for self-renewal and multidirectional differentiation (Rueger and Androutsellis-Theotokis, 2013), although further research is needed to fully understand their involvement (Saha et al., 2012). Inhibiting PTEN expression was shown to increase the survival and proliferation of mesenchymal stem cells in myocardial infarction (Feng et al., 2020), while the proliferation of NSCs and neural progenitor cells is significantly increased following PTEN deletion. Thus, the study of molecular mechanisms that affect NSC proliferation and differentiation is crucial to promoting the repair of neural function.

PTEN is the first tumor suppressor gene known to encode a protein with phosphatase activity. It plays an important role in a variety of diseases by affecting cell proliferation, differentiation, apoptosis, and metabolism, and achieves its physiological effects by interacting with a series of downstream effector molecules (Kuchay et al., 2017). mTOR is one such signal molecule in the PTEN signaling pathway, which is activated through phosphorylation and mediates a series of downstream molecules to promote the synthesis of cellular proteins and cell growth (Yoon and Chen, 2008).

Bpv(pic) is a compound that changes the structure and inactivates the cysteine residues within the catalytic region of protein tyrosine phosphatases, including PTEN. Therefore, we used bpv(pic) as a PTEN inhibitor to investigate its effects on NSC proliferation and differentiation (Mak et al., 2010; Zhang et al., 2017). Bpv(pic) was previously shown to significantly enhance NSC proliferation using a mechanism involving activation of the Akt/mTOR signaling pathway (Zeng and Zhou, 2008; Li et al., 2009). In the present study, we observed a significantly higher number of NSCs after bpv(pic) treatment compared with the control. Additionally, we detected significantly increased expression of PTEN and mTOR in NSCs treated with bpv(pic). This increase in mTOR reflects inhibition of the action of PTEN and an increase, rather than a corresponding decrease, in PTEN expression itself. Because bpv(pic) did not interfere with PTEN expression at the molecular level, bpv(pic) combined with downstream molecules of PTEN, leading to positive feedback that increased PTEN expression (Que et al., 2007; Winbanks et al., 2007)^.^ After bpv(pic) treatment, downstream pathways were activated to increase the expression level of mTOR and affect cell proliferation and differentiation. In nerve cells, the function of mTOR must be maintained within a certain range to promote cell differentiation. However, there is currently no consensus on whether inhibiting PTEN to increase mTOR expression Chappell et al., 2011 promotes or inhibits cell differentiation. 

Our findings suggest that bpv(pic) inhibits the expression of PTEN and promotes the migration and differentiation of NSC into neurons, thus enhancing the repair of central nervous system injuries. This should be explored in future work to investigate potential treatments of central nerve injury.

## Conclusion

In summary, the PTEN inhibitor bpv(pic) promoted the proliferation of NSCs and their differentiation into neurons to some extent. This demonstrates the potential of bpv(pic) to be used in the recovery and treatment of CNS injuries.

## Data Availability

The original contributions presented in the study are included in the article/supplementary material, further inquiries can be directed to the corresponding author.
